# Caries of the Teeth, Its Causes, and a Limited Comparison between the Liability of Different Persons to This Disease

**Published:** 1855-07

**Authors:** Frederick H. Rehwinkel


### ARTICLE V.

*Caries of the Teeth, its Causes, and a Limited Comparison between the Liability of different Persons to this Disease.* By FREDERICK H. REHWINKEL, D. D. S.

CARIES of the teeth, according to Prof. Harris, is "a chemical decomposition of the earthy part of any portion of a tooth, accompanied by a complete disorganization of the animal framework of the affected part."

It is a fearfully insidious and destructive enemy. There is no mouth too pretty into which it does not insinuate itself, no teeth too white and pearl-like upon which it does not fasten its hold; indeed here it is that we most frequently find it, surely and silently engaged in its work of destruction. It is also much more common among the wealthy, who indulge in an indolent and luxurious mode of life, than those in humbler circumstances, who are obliged to exercise constantly and live frugally.

In its attacks it is sometimes exceedingly slow, and as it were systematic; again it is more bold and rapid in its course. This depends, however, entirely upon the nature of its victims. The work of destruction is much sooner completed in teeth of a soft and delicate texture, and which are transparent in appearance, than those of a structure more dense, and to the eye yellowish and very opaque.

The whole crown of a tooth is subject to the attack of caries.

The points where it is most likely to be found, are the depressions in the grinding surfaces of the molars and bicuspids, and the approximal surfaces of all the teeth; also where an imperfection in the enamel may chance to exist. The color of caries may vary from white to black, and depends upon several circumstances, "perhaps some peculiar modifications of the agents upon the presence of which the disease is dependent."

Caries may attack the teeth, and progress very far, before the person so affected is at all aware of its presence. Its detection in the early stages is often difficult even for the practiced eye; and unless a dentist is in the habit of carefully examining every little bluish, brownish, or yellowish spot, observable on the enamel, a great deal may escape his notice, and before much time has elapsed, these germs of caries may give sorrowful proof of their nature. In the approximal surfaces of the teeth, it is often impossible, by reason of the crowded condition of these organs, to ascertain the existence of this disease, until it makes itself known by the entire disappearance of the enamel (as far as its influence has extended) and a perforation into the bony structure of the affected organ.

I have already remarked, that in teeth of a delicate structure, its progress is sometimes exceedingly rapid. Persons possessing such teeth and laboring under constitutional ailments, in which the fluids of the mouth become more viscid, acting thereby more injuriously upon these organs, may, in a short time, unless the difficulty can be removed, lose them entirely. On the other hand, teeth of a stronger texture, even when exposed to the same deleterious influences, will for a much longer time resist the attack of chemical agents, and will not so readily yield to their action.

Caries commences on the external surface of a tooth, and beneath the enamel; from thence it proceeds towards the interior, destroying layer after layer of bone, until the pulp cavity is reached. During this process the enamel may remain firm, and there be no other indication of the presence of the disease save a dark brownish spot. A complete shell is thus formed, which will after a time cave in, thus exposing the whole extent of the cavity. Unless arrested, it will run its course steadily and surely, until in the place of a once beautiful and useful set of teeth, naught remains but an unsightly ruin, which proves an unceasing source of irritation and annoyance to the possessor. Caries during its progress is of much more consequence to the patient, than merely the threatened loss of the affected organ; for when we consider the many constitutional diseases upon which it exerts an exceedingly injurious influence, it will be a matter of no surprise that many good and scientific men, have of late years, devoted their whole time and energies to the discovery of the most successful remedy for this evil. Their efforts have been rewarded, and dental science is now in possession of almost infallible means for the accomplishment of this great object. To return from this slight digression, the medically educated dentist will without hesitation admit, that in many cases of dyspepsia and hysteria, and also in numerous other affections, the true cause (although upon the first view it may seem far from the truth) may frequently be traced to dental caries, and its influence. In regard to the causes of the disease in question, many theories have been from time to time advanced. Of these will I now speak.

First, the chemical theory.

Secondly, the vital.

Thirdly, the chemico-vital; the recent theory of Lintot, viz. the endosmotic, is similar to the first.

Many of the French writers express their concurrence with the first named theory, some, however, in a manner quite vague and undefined. Some express their belief that caries of the teeth is produced by external chemical agents, such as vitiated saliva, particles of food remaining between the teeth or in their interstices, acids, and finally, a corrupted state of the fluids which furnish nourishment for these organs.

It was for a long time the favorite theory of the English authors that inflammation of the dentine, was the immediate cause of caries; one of them ought to have discovered an identity between the teeth and other bone, and formed the conclusion that diseases of the teeth were analogous to those of the other bones. Time and recent observations have proved this to be an error. There are no less than six distinct points of difference between the teeth and other bone.*

We come now to the consideration of the first or chemical theory. This is the most rational of any as yet known, and is, I believe, the one most generally accepted. That caries of the teeth is directly produced by external chemical agents, can be supported by facts, to substantiate which, the most undeniable proof can be produced.

Fluids of the mouth, especially the mucous, when in a vitiated condition are capable of penetrating the enamel of teeth, and decomposing the dentine, when said teeth are possessed of only the ordinary density. To prove this, it is only necessary to insert an artificial set of teeth, made of the crowns of human or animal teeth, into a mouth, in which the fluids are in the condition above described; and the result will be, that decay or caries with all its characteristics will soon manifest itself. Teeth also, when made of bone or ivory, are readily decomposed. These artificial substitutes are certainly not endowed with vitality, and yet are destroyed in the same manner as the natural organs; thus plainly showing that the fluids of the mouth, through the influence of chemical agents alone decompose the material of which they are made—and that caries of the teeth is entirely independent of any vital action. Prof. Harris very justly concludes, "that if all the functional operations of the body were always healthily performed, this disease would never occur;" and he reasons in this way, "that the alkalinity of the saliva would be sufficient to completely neutralize the acidity of the mucous fluids of the buccal cavity, as well as any other acids which might be generated in the mouth."

The second, or vital theory is, I think, successfully refuted by the explanation and proofs of the first, or chemical theory. We will, therefore, dismiss it without further consideration.

In regard to the chemico-vital theory, I will only say, that when inflammation exists in the bony structure of a tooth, we

* See Handy's Anatomy, page 247.

may reasonably suppose, that the susceptibility of the diseased organ to the influence of external agents, is much increased, but it can scarcely be considered as an exciting cause of caries.

We will now consider the indirect causes of dental caries. They are numerous, and might be divided into local and constitutional; among the former, all foreign bodies, such as deposition of salivaay calculus upon the teeth, irregularity in their arrangement, too great a pressure against each other, badly inserted artificial teeth, and every thing which may act irritatingly upon the alveolar and dental membranes, or gums.

Among the constitutional causes which are concerned in promoting caries, I might name for instance, a scrofulous, syphilitic or mercurial diathesis of the system; all inflammatory diseases which may occur during the formation of the teeth, and also an improper nutrition at this time. These two last points I consider of vast importance, for it is at this period of life that the general state of health most reflects upon the whole organism, and especially upon the teeth, even to such a degree as to produce the different imperfections in their structure and physical characteristics. This is still more plainly observed, when acute and inflammatory diseases attack an infant during the process of ossification of the teeth; if the constitution of the child, independently of these diseases, is in a good condition, it will probably be found that only *those* teeth, which at that particular time were ossifying, present the marks of the morbid influence upon them. This is generally seen upon pairs, and may consist in an imperfection of the enamel, or a contraction of the crowns of the teeth; in which case the grinding surfaces of the molars and bicuspids will exhibit deep furrows, which form an excellent receptacle for particles of food and fluids of the mouth; thereby exposing these organs in their weakest points to the influence of chemical action, and thus rendering them exceedingly liable to be attacked by caries. If isolated diseases are, during this period, capable of exerting such injurious influences upon the physical conformation of single teeth, or pairs, how much more prejudicial must be those causes which have their origin in a constitutional derangement of some of the functions of the body! It is evident that in such cases, the whole dental apparatus, is, to a greater or less extent, seriously affected, even before the emancipation of the teeth from the gums.

Another great evil is improper nutrition during the formation of the teeth. Thousands of cases occur in our midst, in which the nutrition, from fetal existence to adult age, is almost wholly contrary to that which nature designed. There is in my opinion too little stress laid on this point. To quote the words of an intelligent author upon this subject, and in whose views I fully concur: "It is rare, if not impossible, to find a mother who entertains the slightest opinion founded upon reason, of the food which should form her diet, before and during preg nancy, or who apprehends the least consequence to her offspring from a gloated appetite which is often so vulgarly and foolishly encouraged." The development of the teeth commences soon after conception, and is one of the greatest and most active labors of nature. The writer above referred to, goes on to say, "the remarkable and intimate relationship between the child and its parent, renders their well-being inseparable, and greatly subject to corresponding changes. The influences affecting the mother must be received by the child, and indulgences or habits which, under ordinary circumstances are, to say the least, doubtful to her, must inevitably tend to affect the child injuriously. No longer can the fact be questioned, that improper kinds of diet, and irregularities of living in the mother, produce the most baneful effects upon the unborn child, resulting in the birth of weak and debilitated children, possessing, as a natural consequence, a defective dentine."

After birth, the infant is still for a time altogether dependent upon its mother for nourishment, and it is, alas, too often found that she, owing to an enfeebled constitution is wholly unfit to perform a mother's office towards her offspring. Yet children are nursed under these circumstances. Perhaps one reason is, that in this country good and healthful nurses are not easily procured. Another is, that mothers generally love to care, themselves, for their little ones, and will reluctantly consent to have their places usurped by others. It is different in Europe, the belief among the higher classes has long been, that, the nursing of children would tend to diminish the beauty and freshness of the mothers; therefore, no sooner are they born than turned off to an hireling, even when the parent is perfectly robust and healthful. In such cases it is a cruel wrong to the infants. On the contrary, the women of this country, so far from sharing in this opinion, in many cases insist upon nursing their own offspring, when it would be actually advisable for them to transfer the duty to another, physically better fitted for its performance.

Children nursed under these circumstances must necessarily be feeble and delicate, and as at this time the development of the whole system goes on more rapidly, proportionately, than at any other period of life, it follows that the whole organism is more or less impaired, but particularly the teeth.

When the time has arrived to discontinue nursing, and gradually accustom the stomachs of infants to the reception of more substantial food, it too often happens that they are at once initiated into all the luxuries of the table, and allowed to eat whatever they may happen to fancy, without any regard to its being a proper article of food. Again, if they have the good fortune to be possessed of parents who are wise and judicious in this respect, there is still a formidable danger menacing them, in the form of over indulgent grand-parents, who are but too easily coaxed, to secretly supply what papa forbids. Finally to sum up, we must include all those people who are in the habit of proving their affections to children, by feasting and overfeeding them on rich cake, nuts, candy, &c., and in fact almost every thing but that which is healthful and proper. This state of things is much to be lamented, since it is in direct opposition to the establishment of good health; and particularly will the denture be injuriously influenced, and its susceptibility to morbid impressions permanently fixed, continuing as long as any portion remains.

This subject is so clearly understood by every one who has even a slight knowledge of, and experience in, general medicine, that I deem it unnecessary to dwell longer upon it, and will close, after endeavoring to draw a limited comparison between the liability of different individuals to caries of the teeth.

I shall commence with this country. It is a generally acknowledged fact, that the women of America, are, as a class, the most beautiful and graceful of any in the world; and it is also a melancholy fact, that dental caries is more commonly found among them; and is increasing rapidly, and to an alarming extent. This statement may appear exaggerated, but good authority can be produced for its support. I do not mean to be understood that this disease is alone confined to the females of this country, but the males are not so subject to its influence.

The luxurious mode of life indulged in by a great portion of the inhabitants of the United States, will partly explain the above assertion. There is perhaps no other country in the world in which it is so general a thing. Only the very poorest, are ignorant of luxuries which have, to the mass, become almost actual necessities. Those who are obliged to labor day by day for the coarse and substantial food which forms their diet, are, from the fact that they are compelled to exercise proportionately to the character of the food by which they subsist, possessed of better health and constitutions than those occupying a more elevated sphere of life.

It is the great fault of the American people, (if I may be permitted so to express myself) to carry every thing to excess. An able American author says, "fashions, both in dress and manners, imported from France will not suffice; they must be Americanized, which means, pushed a little beyond the original." While following and exceeding the Europeans in too much indulgence in this respect, they neglect imitating them in the only thing which would be in the least beneficial, viz. their habits of frequent and healthful exercise in the open air.

The climate, in my opinion, exerts, indirectly, an influence in producing caries. This is in a measure proved by the fact that persons living in some provinces of France are not as liable to the disease as others in different portions of the same country. It has also been observed, that foreigners coming from more equable climates to this country, become more subject to this affection. Some of the northern parts of Europe; Norway, Sweden and Russia, are but little acquainted with caries of the teeth. Of course I have only reference to that class of people who occupy a middle station in life. The higher classes, there as well as here, are proportionately affected by this disease. In some parts of Germany, the inhabitants have good and durable teeth, and the services of the dentist are only required for a limited number. I might say much more on this subject, but will desist; all may be summed up in this one thing: that among those nations or parts of them, in which the primitive, rational, and simple modes of life of our forefathers, are still adhered to, caries of the teeth is comparatively a thing of rare occurrence.

---

